# Configurational paths to turnover intention among primary public health workers in Liaoning Province, China: a fuzzy-set qualitative comparative analysis

**DOI:** 10.1186/s12889-024-17881-8

**Published:** 2024-02-05

**Authors:** Xueying Li, Chenxin Yang, Libing Liu, Yuanlu Ding, Jianchun Xue, Jiani He, Hui Wu, Li Liu

**Affiliations:** Department of Social Medicine, School of Health Management, China Medical University, No. 77 Puhe Road, Shenyang North New Area, Shenyang, Liaoning 110122 China

**Keywords:** Turnover intention, Primary public health workers, Center for disease control and prevention, Configuration, Fuzzy-set qualitative comparative analysis

## Abstract

**Background:**

A stable public health workforce plays an indispensable role in the realization of the goal of health for all. However, there is an exodus of public health workers from the Centers for Disease Control and Prevention (CDC). Given the limited evidence on the mechanisms shaping turnover intention (TI) among public health workers, the study aims to investigate the triggering mechanisms of high and low turnover intention by combining job demands, job resources, and personal resources through a set theory perspective based on the Job-Demand-Resources (JD-R) model.

**Methods:**

The cross-sectional study was conducted from September 7 to 18, 2020 at district (county) level CDC in Liaoning Province, China. A total of 584 public health professionals were included. Overcommitment, effort, social respect, occupational identity, job rewards, self-efficacy, and psychological resilience were included in the study as configuration factors. The data were gathered through an online questionnaire and were analyzed using multiple regression and fuzzy-set Qualitative Comparative Analysis (fsQCA).

**Results:**

Social respect (*B* = -0.682, *P* < 0.001), occupational identity (*B* = -0.168, *P* < 0.001), and effort (*B* = 0.114, *P* < 0.001) were associated with turnover intention. Five configurations for high turnover intention and five for low turnover intention were obtained through the fsQCA, with occupational identity and effort playing an essential role in all pathways. Moreover, the configurations for low turnover intention are not the antithesis of the configurations for high turnover intention.

**Conclusion:**

Managers should synthesize the combined effects of factors when implementing interventions and formulating policies. Given the vital role of occupational identity and effort, mechanisms for the rational distribution of work to avoid excessive efforts and measures to promote occupational identity should be implemented to reduce the turnover intentions of primary public health workers and encourage their intention to stay.

**Supplementary Information:**

The online version contains supplementary material available at 10.1186/s12889-024-17881-8.

## Background

The Centers for Disease Control and Prevention (CDC) are specialized public health agencies in China. CDC at the national, provincial, municipal, and county (district) levels form a four-tiered system [1]. CDC in China is experiencing an outflow of human resources [2]. The turnover leads to a considerable loss of public resources for education and training. It also increases the workload of the remaining workforce and worsens their working conditions. Turnover intention (TI) is considered to be the final psychological state of a person who intends to leave an organization or position at a specific time [3, 4]. Employees with TI may quit their jobs when an external opportunity arises [3]. TI is considered the most reliable predictor of turnover and more worthy to study than turnover [5]. Therefore, understanding the key factors and configurational paths of TI can not only mitigate CDC personnel turnover, but also promote the improvement of public health system.

The Job Demands-Resources (JD-R) model has been widely applied to explore the influencing factors of TI in various occupational groups worldwide. The JD-R model divides job characteristics into job demands and resources that impact critical job outcomes [6]. Job demands are the physical and psychological efforts or costs required from employees. Job resources refer to the material, psychological, and emotional support or assistance that promotes personal development and achievement of work objectives [6]. Employees can be under tremendous work stress due to high job demands. It may ultimately lead to unhealthy organizational outcomes (e.g., turnover) [7]. Adequate job and personal resources can buffer the negative impact of excessive job demands on employees [8], but their physical and mental resources can be exhausted by the constant over-demanding nature of work tasks [7, 9]. Employees will decrease work commitments to preserve their resources when lacking resources [9]. Meanwhile, the motivation to acquire resources will be triggered, leading to their TI [9]. Previous research has shown that job demands and resources are directly related to TI and can indirectly contribute to TI through exhaustion and depersonalization [10, 11]. In addition, lack of resources reinforces the relationship between job demands and exhaustion [10]. Studies have emphasized the versatility and flexibility of the JD-R model. The adaptability of the model among people in different occupations [12–14] and in studies related to TI has also been emphasized [15, 16]. Therefore, the present study used the JD-R model as a theoretical basis for understanding the key factors and configurational paths of TI.

Job demands in the JD-R model were considered to include the time and energy (effort) or the emotional investment (overcommitment) expended on the job [17]. Heavier workloads and more work-life interruptions are strong predictors of TI, and also lead to can lead to higher levels of burnout [15]. Individuals with overcommitment have a frequent urge for recognition and success [18]. It further increases effort and may be related to high levels of stress [19].

For job resources, after expending the effort, the working population usually expects to be rewarded with financial benefits, job security, workplace respect, or expectations of promotions [20, 21]. When lacking rewards, employees experience reduced job satisfaction and engagement and may become exhausted, ultimately leading to TI [10, 13, 22]. In addition, people in China attach great importance to their self-image constructed by the evaluations of others [23]. Individuals always compare themselves to their peers [20]. Compared to clinicians, the CDC workforce perceives less respect from the community [2]. It may be related to their TI. Occupational identity, a strong predictor of TI, refers to a clear understanding of occupational interests, abilities, goals, and values [24, 25]. Occupational identity was suggested to indirectly influence TI among rural public health providers through job satisfaction [26]. Those with high occupational identity are more able to enjoy themselves at work, which may be critical to retention [27].

Bakker further elaborates on the JD-R model and includes personal resources as an essential component of the model [28, 29]. Personality traits are an integral component of personal resources and are increasingly recognized as the key to the comprehension of TI [28]. Self-efficacy and psychological resilience are recognized as resources helpful in counteracting stress [9, 30]. Individuals with higher self-efficacy and psychological resilience may partially ignore the job demands or view them as exciting challenges [9]. Simultaneously, higher self-efficacy and psychological resilience are associated with a higher intention to stay [31, 32]. Therefore, job demands (effort and overcommitment), job resources (rewards, social respect, occupational identity), and personal resources (self-efficacy and psychological resilience) were included in the present study to determine if there are explanations for TI among primary public health workers.

The relationship between the above influences and TI has been extensively explored in the physician and nurse. However, research on the primary public health workforce is insufficient, and the relationship between the combined effects of factors and TI also lacks discussion. In addition, current research has focused on clarifying how factors trigger high TI rather than low TI. For these reasons, there is a necessity for further research to clarify the relationship between the internal mechanism of the above factors and the TI of primary public health workers. The Qualitative Comparative Analysis (QCA) based on a configurational perspective may support us in addressing these issues and give us a better understanding of TI. QCA was proposed by Ragin in 1987 and is based on set theory [33]. In distinction to traditional linear regression, different configurations or paths leading to the same outcome can be obtained in QCA. Moreover, configurations leading to the presence and absence of the outcome variable are considered asymmetric in QCA [34]. Among the various types of QCA, fuzzy set qualitative comparative analysis (fsQCA) uses the fuzzy set scale, which is more applicable to variables with multiple categorical and continuous variables. It avoids classifying study cases as "fully in" or "fully out" and has a broader range of applications [35]. Therefore, the main aim of this study is to provide a theoretical basis for the proposal of related policies by exploring the different configurations that contribute to the TI of primary public health workers and the role of each influencing factor in the configurations using the regression model and fsQCA.

## Methods

### Participants and procedures

It was a study with a cross-sectional observational design. The study was conducted from 7 September 2020 to 18 September 2020 and was supported by CDC managers.

The studies involving human participants were reviewed and approved by the Human Experimentation Ethical Review Committee of China Medical University (YD2020018). Participants gave informed written consent to be involved in this study. The privacy rights of the participants were respected.

We distributed electronic questionnaires to the CDC public health workers at the county (district) level in Liaoning Province through the Wenjuanxing platform. In total, 746 public health workers who volunteered to participate in the survey completed the questionnaire. After excluding the questionnaires with logical contradictions and missing values, 584 valid questionnaires were included, with an effective response rate of 78.3%.

### Measures

To avoid complicating the results with too many conditions, we refer to the criterion of including 3–7 condition variables [36]. A total of seven configurational factors of overcommitment, effort, social respect, occupational identity, job reward, self-efficacy, and psychological resilience were included.

### Sociodemographic characteristics

The sociodemographic characteristics of the respondents included biological sex, age, marital status, education level, and income.

### Social respect

Social respect in this study was self-reported by respondents. Respondents were asked: “How well do you think the community recognizes and respects the profession that you are in?” The answer ranged from 1 (very low) to 5 (very high).

### Occupational identity

The Occupational Identity Scale (OIS) has 10 items and was first developed by Tyler and McCallumand, and the Chinese version was used in this study [37]. The scale was assessed on a five-point Likert scale from 1 (not at all) to 5 (fully). The total score ranged from 10–50, with higher scores indicating a stronger sense of occupational identity. The OIS has been widely used in China with good reliability [38, 39]. The Cronbach’s α coefficient for OIS in this study was 0.948.

### Self-efficacy and psychological resilience

The Chinese version of the Resilience subscale and the Self-efficacy subscale of the 24-item Psychological Capital Questionnaire (PCQ-24) were used to measure the psychological resilience and self-efficacy of CDC public health workers. The PCQ-24 was developed by Luthans [30] and is widely used in China [40]. Higher scores indicate greater psychological resilience or self-efficacy. Both subscales contained six items. Each item was scored on a scale of 1 (strongly disagree) to 6 (strongly agree). The total score of each subscale ranged from 6–36. In this study, Cronbach’s α coefficients for the self-efficacy and psychological resilience subscales were 0.927 and 0.937, respectively.

### Effort, overcommitment, and reward

The Effort-Reward Imbalance Questionnaire (ERIQ) was developed by Siegrist in 1996 [17], and the Chinese version with good reliability was used in this study [41]. The questionnaire has 23 items and consists of 3 dimensions: effort, reward, and overcommitment. The effort dimension consists of 6 items, assessed on a 5-point Likert scale (from 1 disagree to 5 strongly agree) with scores ranging from 6–30, and higher scores indicate more effort. The reward dimension consists of 11 items assessed on a 5-point Likert scale with a total score ranging from 11–55. A higher score means more job reward. The overcommitment subscale consisted of 6 items assessed on a 4-point Likert scale from 1 completely disagree to 4 completely agree. Scores on this subscale ranged from 6–24 with higher scores indicating more severe job overcommitment. The Cronbach’s α coefficient of ERIQ in this study is 0.728. In addition, the Cronbach’s α coefficients for the effort, reward, and overcommitment subscales were 0.867, 0.809, and 0.725, respectively.

### Turnover intention

The Chinese version of Turnover Intentions Questionnaire (TIQ) with good reliability was used [40]. The TIQ was first developed by Michael and Spector [42]. The scale has six items and three dimensions, which are the likelihood of quitting your current job (items 1 and 6), the likelihood of finding another job (items 2 and 3), and the probability of obtaining other jobs (items 4 and 5). The current study used a reverse scoring scale of 1–4 (from 1 often to 4 never) to assess the TI. A total score ranging from 6–24 was obtained. The higher the total score, the stronger the TI. The Cronbach’s α coefficient of TIQ in the present study was 0.835.

### Statistical analysis

SPSS 21.0 for Windows (IBM, Asia Analytics, Shanghai) and fsQCA4.0 were used for analyses. The results for the sociodemographic characteristics of the respondents were expressed as the mean and standard deviation for continuous data and the percentage for categorical data. Multiple linear regression models were performed to test the linear relationship between multiple independent variables and TI. FsQCA was used to analyze configurations. Following the fsQCA analysis process, the data were calibrated and subjected to necessity and sufficiency analyses, and finally, robustness tests were performed.

## Result

### Descriptive statistics

A total of 584 CDC public health workers were surveyed. The average age of them was 44.48 ± 9.65 years. 415 (71.1%) of respondents were female. 36.3% of the respondents had an income of 3,000–4,000/month, and 65.2% had a bachelor's degree. 14.2% of respondents felt that their profession was poorly respected and recognized by residents. The mean scores of occupational identity, self-efficacy, psychological resilience, effort, reward, overcommitment, and TI for CDC public health workers were 39.69 ± 7.48, 29.21 ± 4.34, 29.19 ± 4.53, 18.67 ± 5.76, 26.73 ± 8.41, 15.69 ± 2.71, and 11.42 ± 3.96, respectively. More details can be found in the supplementary material (Table S1).

### Multiple regression models

Social respect (*B* = -0.682, *P* < 0.001) and occupational identity (*B* = -0.168, *P* < 0.001) are negatively associated with TI. Occupational identity showed a stronger negative association than social respect (social respect: *β* = -0.152, occupational identity: *β* = -0.318). Effort (*B* = 0.114, *P* < 0.001) demonstrated a positive and significant beta. More details are provided in Table 1.

**Table 1:** The results of the multiple linear regressions

Variables	*B*	S.E	*β*	*t*	*P*
Constant	16.323	1.494	-	10.929	** < 0.001**
Social respect	-0.682	0.178	-0.152	-3.825	** < 0.001**
Occupational identity	-0.168	0.024	-0.318	-7.095	** < 0.001**
Self-efficacy	0.079	0.060	0.086	1.315	0.189
Psychological resilience	-0.042	0.059	-0.048	-0.719	0.472
Effort	0.114	0.032	0.166	3.613	** < 0.001**
Reward	-0.007	0.018	-0.015	-0.395	0.693
Overcommitment	0.003	0.064	0.002	0.052	0.958

*B* estimated coefficient, *S.E.* standard error, *β* standardized regression coefficients

### Fuzzy set qualitative comparative analysis model (fsQCA)

#### Calibration

The data were calibrated with P5 (fully out), P50 (crossover), and P95 (fully in) for continuous variables [43]. For social respect, a five-valued fuzzy set was created using an indirect calibration method [44]. In fsQCA, cases with a membership score of 0.5 are difficult to analyze. Therefore, we added a constant of 0.001 to cases where the configuration factor was lower than the full-membership score of 1 [45]. Statistical descriptions and calibration values of the studied variables are summarized in Table 2.

**Table 2:** Statistical descriptions and calibration values

	Occupational identity	Self-efficacy	Psychological resilience	Effort	Reward	Over-commitment	TI
M	39.69	29.21	29.19	18.67	26.73	15.69	11.42
SD	7.48	4.34	4.53	5.76	8.41	2.71	3.96
Min	10	12	12	6	11	6	6
Max	50	36	36	30	55	24	24
Calibration values							
P_5_	26.25	22.00	21.25	9.00	15.00	11.00	6.00
P_50_	40.00	30.00	30.00	19.00	25.00	16.00	11.00
P_95_	50.00	36.00	36.00	28.00	49.00	20.00	18.00

*M* mean, *SD* standard deviation, *Min* minimum, *Max* maximum, *P5* 5th percentile, *P50* 50th percentile, *P95* 95th percentile

#### Necessity analysis

Based on previous studies, a consistency benchmark of 0.90 was used to determine the necessary conditions (The result cannot be produced without this condition.) for the outcome [46]. According to the results derived from Table 3, neither high TI nor low TI has the necessary conditions.

**Table 3:** Results of the necessity analysis of high and low TI

configuration factors	High TI			Low TI	
	Consistency	Coverage		Consistency	Coverage
Social respect	0.624	0.640		0.692	0.751
~ Social respect	0.757	0.699		0.668	0.652
Occupational identity	0.590	0.549		0.756	0.744
~ Occupational identity	0.724	0.738		0.542	0.583
Self-efficacy	0.592	0.612		0.645	0.704
~ Self-efficacy	0.714	0.655		0.644	0.625
Psychological resilience	0.597	0.606		0.663	0.712
~ Psychological resilience	0.716	0.668		0.633	0.624
Effort	0.708	0.694		0.583	0.604
~ Effort	0.596	0.575		0.705	0.719
Reward	0.648	0.658		0.656	0.704
~ Reward	0.709	0.661		0.681	0.672
Overcommitment	0.680	0.678		0.602	0.633
~ Overcommitment	0.632	0.600		0.694	0.697

~ refers to negation. In this study, ~ X was considered to be a lower level of X

#### Sufficiency analysis

The consistency threshold was set to 0.8 in the sufficiency analysis [47]. The Proportional Reduction of Inconsistency (PRI) was set to 0.65 [48, 49]. We set the frequency threshold to 5 to ensure that at least 80% of the cases were retained in the analysis [50]. Ultimately, 82.5% of cases were kept. To obtain all possible configurations, the study conducts the counterfactual analysis without making assumptions about the direction of configuration factors [51]. Parsimonious, complex, and intermediate solutions are available in fsQCA. Configuration factors that appear in parsimonious and intermediate solutions are defined as core conditions, which cause a significant effect on the outcome. Peripheral conditions play an auxiliary role and appear only in the intermediate solutions.

#### Configurations for achieving the high TI

Table 4 shows the five pathways leading to the high TI among primary public health workers. The consistency coefficient of the solution should be > 0.75, and the coverage rate should be > 0.25 [47]. The consistency and raw coverage of each configuration were up to standards, so the results of this analysis can be considered to have good consistency and coverage. 85.2% of the cases that met these five configurations had a low TI. In addition, 48.2% of the cases can be explained through these configurations. Configurations with the same core conditions are considered to be the second-order equifinal configurations. Therefore, two second-order equifinal configurations were obtained.

**Table 4:**
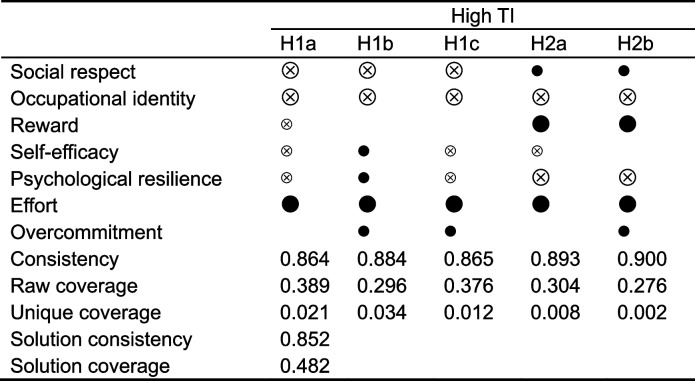
Configurations for achieving the high turnover intention (fsQCA)

*TI* turnover intention; “  ”, presence as a core condition; “  ”, presence as a peripheral condition; “  ”, absent as a core condition; “  ”, absent as a peripheral condition; Blank cells represent ambiguous condition

#### Configurations for achieving the low TI

The five configurations leading to the low TI are shown in Table 5. The consistency and raw coverage of each configuration were up to standards. 85.9% of the cases that met these five configurations had a low TI. In addition, 52.2% of the cases can be explained through these configurations. The five configurations formed a second-order equifinal configuration.

**Table 5:**
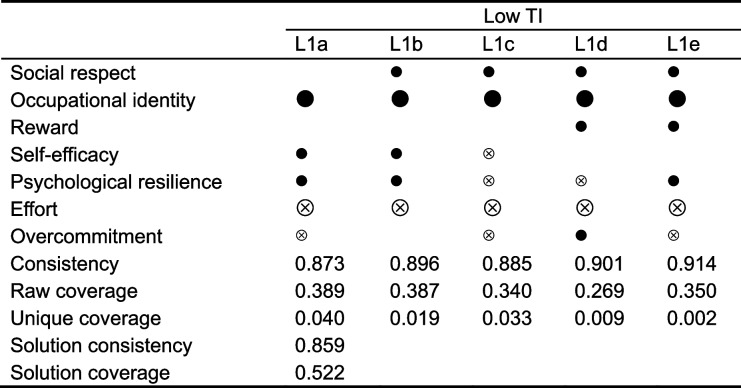
Configurations for achieving the low turnover intention (fsQCA)

*TI* turnover intention; “  ”, presence as a core condition; “  ”, presence as a peripheral condition; “  ”, absent as a core condition; “  ”, absent as a peripheral condition; Blank cells represent ambiguous condition

#### Robustness analyses

We vary the frequency thresholds and calibration strategies to check the robustness of the results. The results are summarized in Table 6. For high TI, variations to the frequency thresholds and anchors produced the same results as the primary solutions. In addition, changing the anchors resulted in a slight decrease in the coverage of the solution. For low TI, the variation of the frequency threshold produced the same results as the primary solution. However, changing the anchors resulted in a new configuration. The new configuration is social respect * occupational identity * self-efficacy * psychological resilience * ~ reward * ~ overcommitment, with occupational identity, ~ reward, and ~ overcommitment as the core conditions. Higher levels of occupational identity are likely to increase the potential for employees to acquire and use knowledge in practice, enabling them to better respond to the demands of their jobs [52]. It might avoid the need to overcommit to their work and make it easier for them to relax. Moreover, adequate personal psychological resources enable them to be more positive in facing work pressure and achieve their own growth and career development [53]. Compensation is a motivating factor, but after a while, it tends to become a factor of dissatisfaction for the employee again [54], hence the emergence of the core condition of “ ~ reward”. The detailed results of robustness analyses are presented in the supplementary material (Table S2 and Table S3 for high TI; Table S4 and Table S5 for low TI). Overall, solutions of the principal analysis remain stable in all robustness tests. Our reported findings can be considered reliable.

**Table 6:** Summary of robustness tests

Outcomes		Calibration anchors/frequency thresholds	Number of configurations	Solution consistency	Solution coverage	Configuration differences
High TI	Main analysis	P_5_, P_50_, P_95_ /5	5	0.852	0.482	-
	Changing frequency thresholds	P_5_, P_50_, P_95_ /4	5	0.852	0.482	None
	Changing calibration anchors	P_7.5_, P_50_, P_92.5_/5	5	0.851	0.471	None
Low TI	Main analysis	P_5_, P_50_, P_95_ /5	5	0.859	0.522	-
	Changing frequency thresholds	P_5_, P_50_, P_95_ /4	5	0.859	0.522	None
	Changing calibration anchors	P_7.5_, P_50_, P_92.5_/5	6	0.861	0.530	An additional configuration

*TI* turnover intention, *P5* 5th percentile, *P50* 50th percentile, *P95* 95th percentile, *P7.5* 7.5th percentile, *P92.5* 92.5th percentile

## Discussion

This study investigated the linear relationship and configuration path between job characteristics and TI among primary public health workers through regression and fsQCA models, and three main results were drawn. First, the results of the regression analyses indicate that social respect and occupational identity are protective factors for TI, while effort is a risk factor for TI. Second, the fsQCA analysis yielded five configurations for high TI and five for low TI. It is to be noted that configurations with low TI were not opposites of high TI. Finally, occupational identity and effort play significant roles in triggering both high and low TI.

The regression model indicated that occupational identity, social respect, and effort were associated with TI. Similar to our study, occupational identity was associated with the TI of physicians [39]. However, occupational identity is not directly related to Chinese rural public health service workers' intention to leave their jobs but is mediated through job satisfaction [26]. In rural China, public health services are usually provided by rural doctors on a part-time basis. Differences in work assignments may have contributed to the inconsistent results of the study. Studies conducted on teachers, nurses, and physicians demonstrated similar results to the present study: effort due to time and physical workload was associated with TI [55–57]. In addition, findings from studies conducted within the direct care workforce support our view that respect from the community is an important influence on TI [58].

For the sufficiency analysis in fsQCA, all five pathways that lead to high TI reflect an imbalance between job resources and demands. H1a, H1b, and H1c demonstrate that low social respect, low occupational identification, and high effort as shared core conditions performed an essential role in TI. It matches the results of the regression model. This outcome may be attributed to the increasing burden of chronic diseases and frequent public health emergencies in recent years. The consequent increasing workload and job risks for primary public health workers can be burdensome. In addition, according to the social comparison theory, employees always tend to compare themselves with people who are similar to them in some self-concepts [59]. CDC workers receive less social respect than clinicians [2], and this comparison may lead to increased psychosocial stress through a sense of relative deprivation [60]. It ultimately affects their career choices. Additionally, lower social respect may be associated with poorer occupational identity and indirectly reduce retention intentions through job satisfaction [61, 62].

H1a and H1c illustrate that high job demands can continually deplete the physiological and psychological resources of the employee. They will develop high TI when job resources are insufficient [9, 63]. Previous research has shown that excessive stress affects TI by reducing psychological resilience or self-efficacy [64, 65]. In addition, H1b suggests that when employees face high job demands but lack appropriate job resources, even the presence of positive personal psychological resources still produces high TI. Excessive job demands may be seen as a challenge. Employees with adequate personal resources can overcome difficulties to achieve their goals better [9]. However, when the effort required to overcome hardships exceeds a certain level, even people with sufficient psychological resources may feel burdened, causing them to develop TI.

Low occupational identity, low mental resilience, high effort, and high rewards were core conditions in H2a and H2b. These two paths may suggest that among employees with low psychological resilience and occupational identity, the drain on physical and psychological resources from excessive job demands cannot be buffered by job rewards. The relationship between TI and job rewards varies depending on the psychological resource sufficiency of the individual. This may be related to different perceptions of low salaries and the relationship between investment and return. Health professionals are motivated by the desire to provide quality care without paying excessive personal costs, according to the Health Professional Wellness Hierarchy [66]. Physical and psychological fitness is recognized as the most basic need in the model, ahead of being respected, appreciated, and well-paid [67]. Previous studies have demonstrated that psychological resilience and self-efficacy can mitigate the impact of work stress on mental health [68]. When psychological resilience and self-efficacy are lacking, excessive stress can be detrimental to mental health and lead to TI [69]. Therefore, compensation may not be a more vital concern than physical and mental health for workers with low personal resources.

The five pathways developed in the sufficiency analysis of low TI suggest an essential role for high occupational identity and low effort. The fact that the configuration of low TI is not the antithesis of high TI indicates that we can’t simply reverse the factors of high TI to derive low TI. L1a and L1b show that low TI is triggered when high occupational identity and low effort are complemented by better personal psychological resources, regardless of social respect, rewards, and overcommitment. Those with high occupational identity are likely to handle work tasks more effectively, so they may be less stressed and happier while working [70]. The low work-related stress was related to increased occupational identity and further enhanced psychological resources [71]. Moreover, occupational identity moderates the relationship between self-efficacy and TI [72]. From another perspective, high psychological resilience and self-efficacy are associated with increased levels of occupational identity and buffer the depletion caused by work stress [73, 74]. A mutually reinforcing good state of affairs seems to have developed. Thus, employees in both configurations had a clearer understanding of their jobs and were better able to handle the tasks, possibly contributing to the low TI.

The L1d configuration suggests that low TI can be elicited if there are sufficient job rewards and no effort is required, even with overcommitment. In this pathway, high overcommitment and high rewards coexist. Previous studies have confirmed that high overload/high reward may be associated with the lowest levels of job dissatisfaction [75]. One possible reason is that the workers who fit this configuration may be more engaged with tasks. It was found that overcommitment and work rewards were positively related to work engagement, while effort was negatively related to work engagement [76]. It seems beneficial for the organization to increase the overcommitment of the employee. However, given the association between overcommitment and severe psychological stress [77], managers should keep tight control of the severity of overcommitment in the practice. The positive impact of overcommitment on organizational behavior should not be achieved at the expense of employee health.

The high level of job resources and the low level of job requirements create an incongruous configuration in L1c and L1e. Over-benefiting from low pay/high rewards can be a potential pitfall in public health workforce development. These employees may lack challenging tasks, resulting in low work engagement [78]. This configuration leads to an overinvestment of work resources even though it is associated with lower TI. Managers should consider adjusting the job demands of employees who fit these configurations to create a high-effort/high-reward format that increases work engagement and results in more positive outcomes [75].

The present study presents several notable advantages. First, as far as we know, this study is the first to investigate the factors influencing TI from a configurational perspective among primary public health workers in China. This study alleviates the lack of research on TI among public health workers. Second, this study combines traditional quantitative methods with qualitative comparative analysis. It facilitates a clearer understanding of the mechanics of TI. Finally, this study explores the factors that trigger low and high TI from the causal asymmetry, providing a new perspective for managers to take effective management measures.

Inevitably, there are some limitations. Firstly, public health workers from other medical institutions are not included (e.g., public health workers in community healthcare centers or rural physicians). Therefore, the study results do not reflect all primary public health workers. Second, the non-probability sampling we used may somewhat hinder the validity of our findings. Third, since the data for this study were derived from self-reports, it inevitably introduces recall bias, and the common method bias may exist. The results of the Harman single-factor test showed that without rotation, eight principal components were extracted with eigenvalues greater than 1, and the maximum one explained 24.40% of the total variance, which is less than 40%. It can be assumed that the common method bias does not significantly affect the measurements [79]. Fourth, this study is cross-sectional and does not consider the role of the time. Future studies could use longitudinal designs or Temporal Qualitative Comparative Analysis (TQCA). Finally, the configuration variables included in this study were limited. Additional work-related factors should be considered to produce more complete results and applied implications. Despite the limitations, this study still expands the research on the TI of public health practitioners.

## Conclusion

All configurations of high TI in this study reflect a job demands/resources imbalance. Therefore, managers should consider adjusting job characteristics to reduce the TI of primary public health workers. Managers should distribute work appropriately and encourage employees to devote their energies to challenging tasks. Recognition of the public health profession among residents should be increased. Moreover, interventions should be provided to strengthen the occupational identity of primary public health workers.

### Supplementary Information

**Additional file 1:** **Table S1.** Demographic characteristics of primary public health workers (*N *= 584). **Table S2.** Results of robustness analysis of changing frequency thresholds in high TI. **Table S3.** Results of robustness analysis of changing anchors in high TI. **Table S4.** Results of robustness analysis of changing frequency thresholds in low TI. **Table S5.** Results of robustness analysis of changing anchors in low TI.

## Data Availability

The data that support the findings of this study are available from the corresponding author (lliu09@cmu.edu.cn) upon reasonable request.

## Abbreviations

CDC: The Center for Disease Control and Prevention
TI: Turnover intention
JD-R: Job-Demand-Resources
QCA: Qualitative Comparative Analysis
fsQCA: Fuzzy-set Qualitative Comparative Analysis
OIS: Occupational Identity Scale
PCQ-24: 24-Item Psychological Capital Questionnaire
ERIQ: Effort-Reward Imbalance Questionnaire
TIQ: Turnover Intentions Questionnaire
PRI: Proportional Reduction of Inconsistency
TQCA: Temporal Qualitative Comparative Analysis
