# Supplementary material for: Configurational paths to turnover intention among primary public health workers in Liaoning Province, China: a fuzzy-set qualitative comparative analysis
Source: BMC Public Health. 2024 Feb 5;24:369. doi: 10.1186/s12889-024-17881-8 (PMC10840158; doi:10.1186/s12889-024-17881-8)
Supplement: Supplementary file 1 — Additional file 1: Table S1. Demographic characteristics of primary public health workers (N = 584). Table S2. Results of robustness analysis of changing frequency thresholds in high TI. Table S3. Results of robustness analysis of changing anchors in high TI. Table S4. Results of robustness analysis of changing frequency thresholds in low TI. Table S5. Results of robustness analysis of changing anchors in low TI. [file 12889_2024_17881_MOESM1_ESM.docx]

**Supplementary** **material**

**Title**: Turnover Intention of Primary Public Health Workers in Liaoning Province and Its Influencing Factors: A fuzzy-set qualitative comparative analysis

**Table. S1.** Demographic characteristics of primary public health workers (*N* = 584).

| Variables | M (SD) / *N* (%) |
| --- | --- |
| **Age** | 44.48 (9.65) |
| **Sex** |  |
| female | 415(71.1) |
| male | 169(28.9) |
| **Marital status** |  |
| Single | 131 (22.4) |
| Married | 453 (77.6) |
| **Education level** |  |
| college and below | 166 (28.4) |
| bachelor’s degree | 381 (65.2) |
| master’s degree and above | 37 (6.3) |
| **Income** |  |
| ≤ 3000 | 125 (21.4) |
| 3000–4000 | 212 (36.3) |
| 4000–5000 | 161 (27.6) |
| > 5000 | 86 (14.7) |
| **Social respect** |  |
| Very poor | 53 (9.1) |
| Poor | 83 (14.2) |
| General | 342 (58.6) |
| High | 87 (14.9) |
| Very high | 19 (3.3) |
| **Occupational identity** | 39.69 (7.48) |
| **Self-efficacy** | 29.21 (4.34) |
| **Psychological resilience** | 29.19 (4.53) |
| **Effort** | 18.67 (5.76) |
| **Reward** | 26.73 (8.41) |
| **Overcommitment** | 15.69 (2.71) |
| **Turnover intention** | 11.42 (3.96) |

*M*, mean; *SD*, standard deviation;

**Table S2.** Results of robustness analysis of changing frequency thresholds in high TI.

|  | High TI | | | | |
| --- | --- | --- | --- | --- | --- |
|  | H1a | H1b | H1c | H2a | H2b |
| Social respect | ⮾ | ⮾ | ⮾ | ● | ● |
| Occupational identity | ⮾ | ⮾ | ⮾ | ⮾ | ⮾ |
| Reward | ⮾ |  |  | ● | ● |
| Self-efficacy | ⮾ | ● | ⮾ | ⮾ |  |
| Psychological resilience | ⮾ | ● | ⮾ | ⮾ | ⮾ |
| Effort | ● | ● | ● | ● | ● |
| Overcommitment |  | ● | ● |  | ● |
| Consistency | 0.864 | 0.884 | 0.865 | 0.893 | 0.900 |
| Raw coverage | 0.389 | 0.296 | 0.376 | 0.304 | 0.276 |
| Unique coverage | 0.021 | 0.034 | 0.012 | 0.008 | 0.002 |
| Solution consistency | 0.852 |  |  |  |  |
| Solution coverage | 0.482 |  |  |  |  |

The frequency threshold was changed (from 5 to 4), but the other settings were consistent with the principal analysis.
TI, turnover intention; “●”, presence as a core condition; “●”, presence as a peripheral condition; “⮿”, absent as a core condition; “⮿”, absent as a peripheral condition; Blank cells represent ambiguous condition.

**Table S3.** Results of robustness analysis of changing anchors in high TI.

|  | High TI | | | | |
| --- | --- | --- | --- | --- | --- |
|  | H1a | H1b | H1c | H2a | H2b |
| Social respect | ⮾ | ⮾ | ⮾ | ● | ● |
| Occupational identity | ⮾ | ⮾ | ⮾ | ⮾ | ⮾ |
| Reward | ⮾ |  |  | ● | ● |
| Self-efficacy | ⮾ | ● | ⮾ | ⮾ |  |
| Psychological resilience | ⮾ | ● | ⮾ | ⮾ | ⮾ |
| Effort | ● | ● | ● | ● | ● |
| Overcommitment |  | ● | ● |  | ● |
| Consistency | 0.864 | 0.885 | 0.863 | 0.895 | 0.902 |
| Raw coverage | 0.363 | 0.267 | 0.358 | 0.285 | 0.250 |
| Unique coverage | 0.024 | 0.033 | 0.015 | 0.011 | 0.002 |
| Solution consistency | 0.851 |  |  |  |  |
| Solution coverage | 0.471 |  |  |  |  |

The calibration anchors were changed from the 5th, 50th, and 95th percentile to the 7.5th, 50th, and 92.5th percentile, but the other settings were consistent with the principal analysis.
TI, turnover intention; “●”, presence as a core condition; “●”, presence as a peripheral condition; “⮿”, absent as a core condition; “⮿”, absent as a peripheral condition; Blank cells represent ambiguous condition.

**Table S4.** Results of robustness analysis of changing frequency thresholds in low TI.

|  | Low TI | | | | |
| --- | --- | --- | --- | --- | --- |
|  | L1a | L1b | L1c | L1d | L1e |
| Social respect |  | ● | ● | ● | ● |
| Occupational identity | ● | ● | ● | ● | ● |
| Reward |  |  |  | ● | ● |
| Self-efficacy | ● | ● | ⮾ |  |  |
| Psychological resilience | ● | ● | ⮾ | ⮾ | ● |
| Effort | ⮾ | ⮾ | ⮾ | ⮾ | ⮾ |
| Overcommitment | ⮾ |  | ⮾ | ● | ⮾ |
| Consistency | 0.873 | 0.896 | 0.885 | 0.901 | 0.914 |
| Raw coverage | 0.389 | 0.387 | 0.340 | 0.269 | 0.350 |
| Unique coverage | 0.040 | 0.019 | 0.033 | 0.009 | 0.002 |
| Solution consistency | 0.859 |  |  |  |  |
| Solution coverage | 0.522 |  |  |  |  |

The frequency threshold was changed (from 5 to 4), but the other settings were consistent with the principal analysis.
TI, turnover intention; “●”, presence as a core condition; “●”, presence as a peripheral condition; “⮿”, absent as a core condition; “⮿”, absent as a peripheral condition; Blank cells represent ambiguous condition.

**Table S5.** Results of robustness analysis of changing anchors in low TI.

|  | Low TI | | | | |  |
| --- | --- | --- | --- | --- | --- | --- |
|  | S1a | S1b | S1c | S1d | S1e | S2 |
| Social respect |  | ● | ● | ● | ● | ● |
| Occupational identity | ● | ● | ● | ● | ● | ● |
| Reward |  |  |  | ● | ● | ⮾ |
| Self-efficacy | ● | ● | ⮾ |  |  | ● |
| Psychological resilience | ● | ● | ⮾ | ⮾ | ● | ● |
| Effort | ⮾ | ⮾ | ⮾ | ⮾ | ⮾ |  |
| Overcommitment | ⮾ |  | ⮾ | ● | ⮾ | ⮾ |
| Consistency | 0.878 | 0.904 | 0.897 | 0.916 | 0.926 | 0.895 |
| Raw coverage | 0.349 | 0.356 | 0.309 | 0.232 | 0.307 | 0.291 |
| Unique coverage | 0.037 | 0.023 | 0.037 | 0.012 | 0.003 | 0.038 |
| Solution consistency | 0.861 |  |  |  |  |  |
| Solution coverage | 0.530 |  |  |  |  |  |

The calibration anchors were changed from the 5th, 50th, and 95th percentile to the 7.5th, 50th, and 92.5th percentile, but the other settings were consistent with the principal analysis.
TI, turnover intention; “●”, presence as a core condition; “●”, presence as a peripheral condition; “⮿”, absent as a core condition; “⮿”, absent as a peripheral condition; Blank cells represent ambiguous condition.
